# Supplementary material for: Alpha-synuclein aggregates are phosphatase resistant
Source: Acta Neuropathol Commun. 2024 May 31;12:84. doi: 10.1186/s40478-024-01785-0 (PMC11141014; doi:10.1186/s40478-024-01785-0)
Supplement: Supplementary file 2 [file 40478_2024_1785_MOESM2_ESM.pdf]

A

Enzyme	Number of Cleavage sites	Positions of Cleavage sites
Trypsin	15	6 10 12 21 23 32 34 43 45 58 60 80 96 97 102
Glutamyl endopeptidase	18	13 20 28 35 46 57 61 83 104 105 110 114 123 126 130 131 137 139

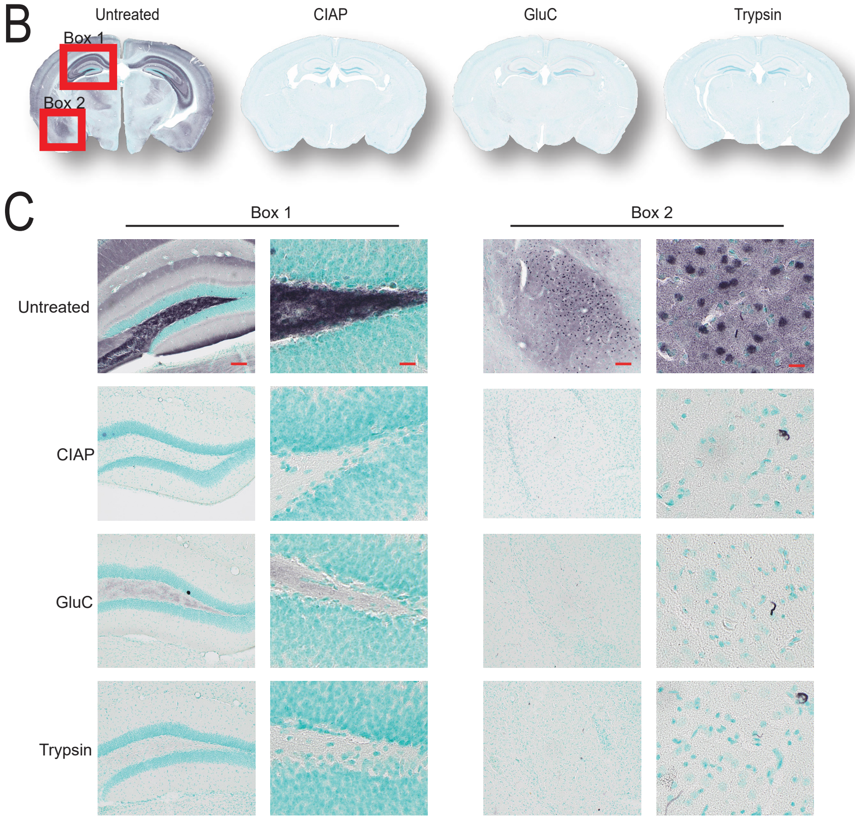

Supplementary Figure 1

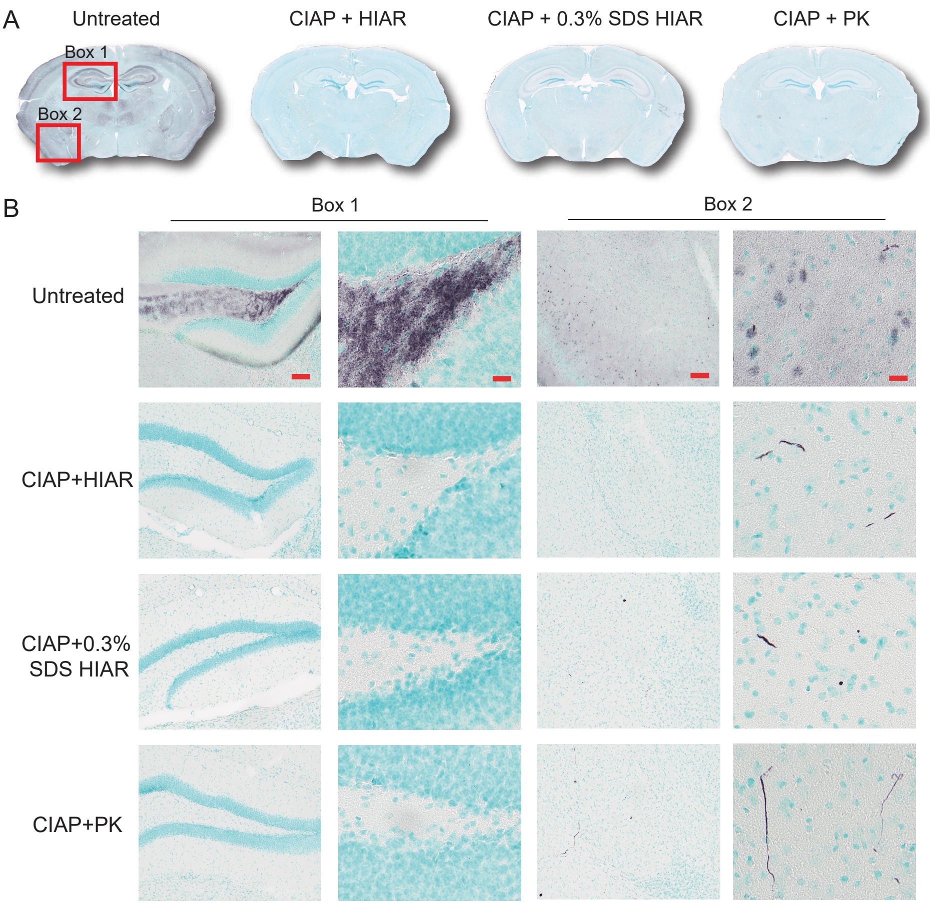

Supplementary Figure 2

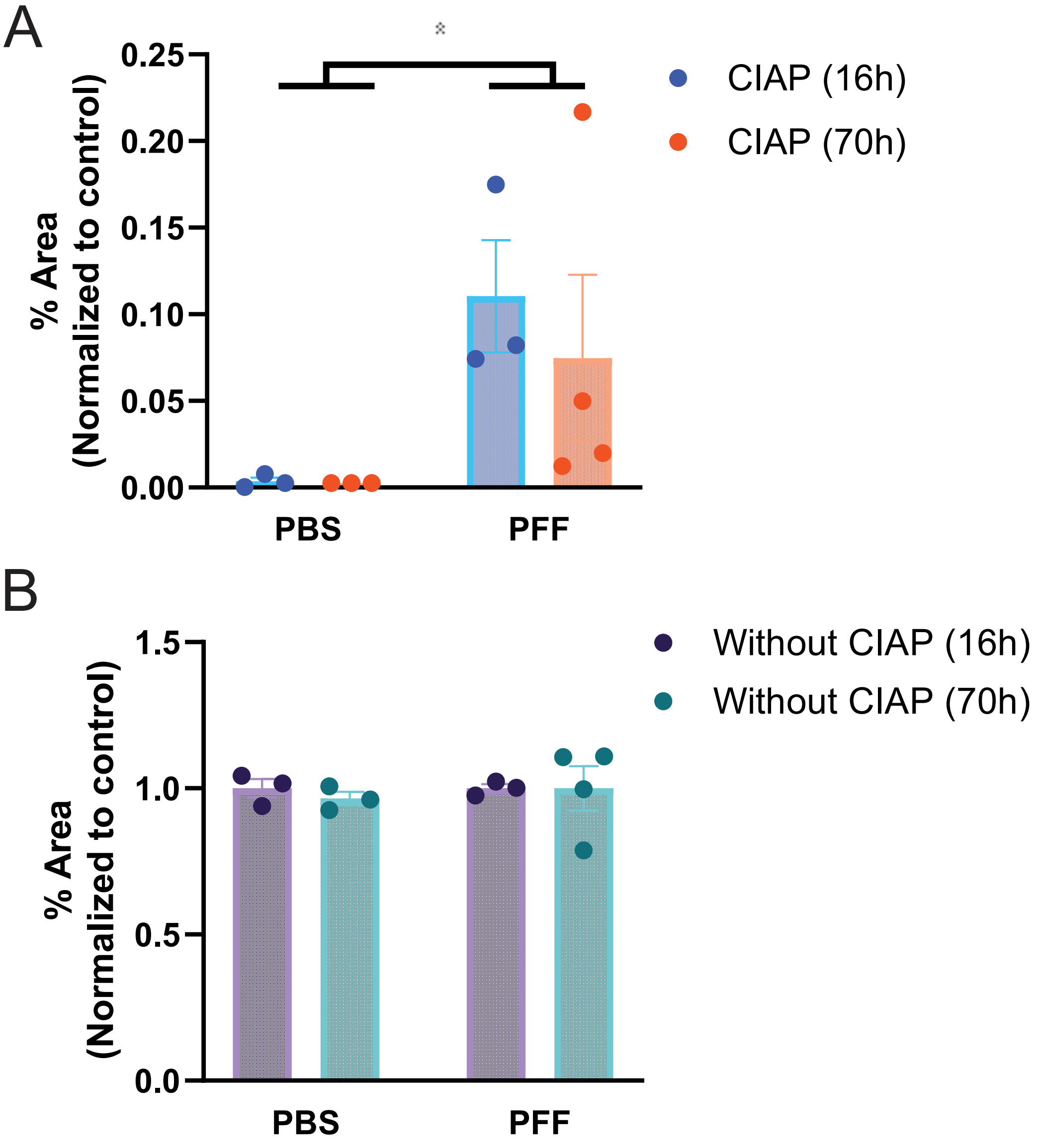

Supplementary Figure 3

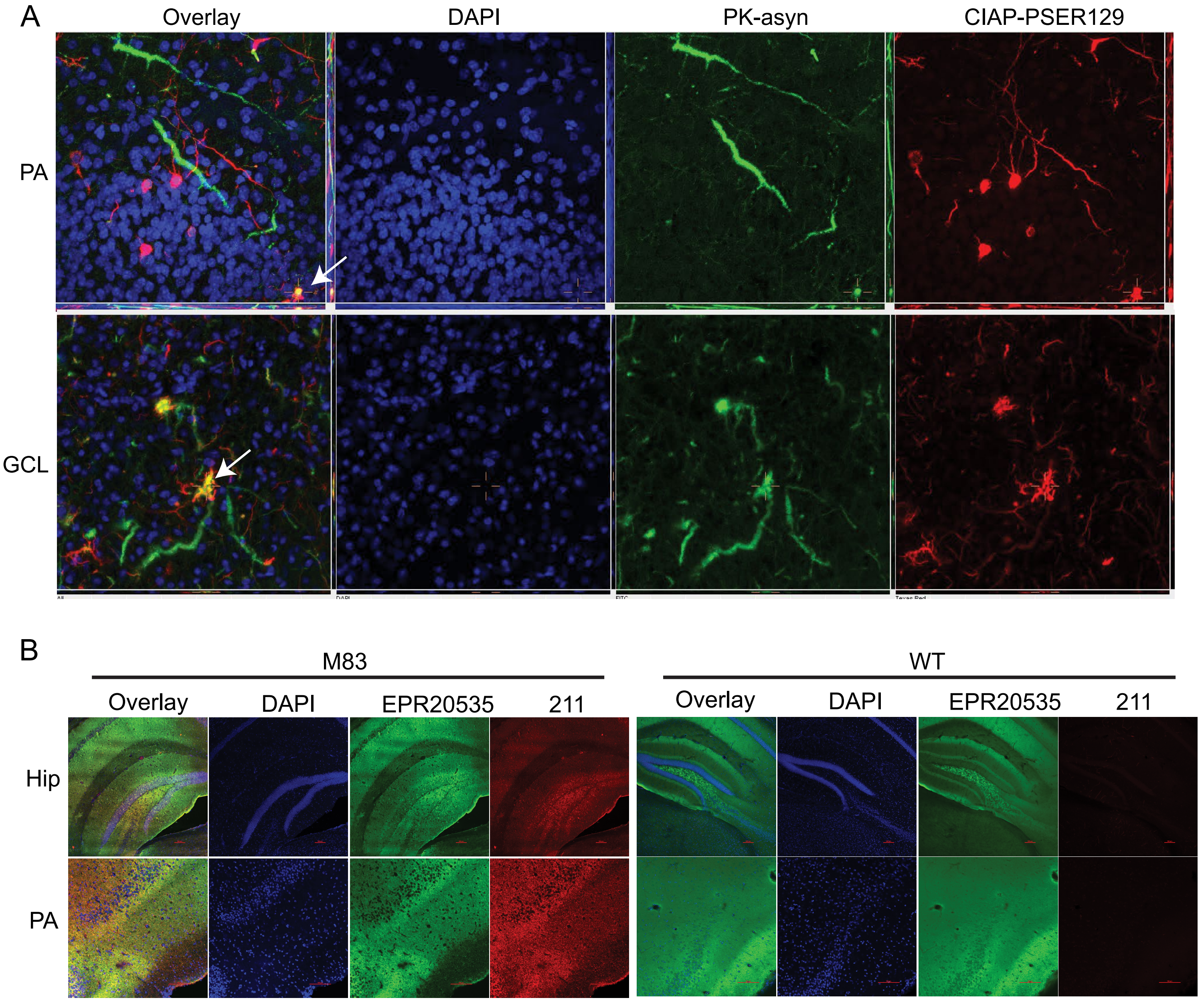

Supplementary Figure 4
